# Structure of a variable lymphocyte receptor-like protein from the amphioxus *Branchiostoma floridae*

**DOI:** 10.1038/srep19951

**Published:** 2016-01-29

**Authors:** Dong-Dong Cao, Xin Liao, Wang Cheng, Yong-Liang Jiang, Wen-Jie Wang, Qiong Li, Jun-Yuan Chen, Yuxing Chen, Cong-Zhao Zhou

**Affiliations:** 1 Hefei National Laboratory for Physical Sciences at the Microscale and School of Life Sciences, University of Science and Technology of China, Hefei Anhui 230027, China; 2 Beihai Marine Station, Evo-devo Institute, School of Life Sciences, Nanjing University, Hankou Road 22#, Nanjing, Jiangsu, 210093, China

## Abstract

Discovery of variable lymphocyte receptors (VLRs) in agnathans (jawless fish) has brought the origin of adaptive immunity system (AIS) forward to 500 million years ago accompanying with the emergence of vertebrates. Previous findings indicated that amphioxus, a representative model organism of chordate, also possesses some homologs of the basic components of TCR/BCR-based AIS, but it remains unknown if there exist any components of VLR-based AIS in amphioxus. Bioinformatics analyses revealed the amphioxus *Branchiostoma floridae* encodes a group of putative VLR-like proteins. Here we reported the 1.79 Å crystal structure of Bf66946, which forms a crescent-shaped structure of five leucine-rich repeats (LRRs). Structural comparisons indicated that Bf66946 resembles the lamprey VLRC. Further electrostatic potential analyses showed a negatively-charged patch at the concave of LRR solenoid structure that might be responsible for antigen recognition. Site-directed mutagenesis combined with bacterial binding assays revealed that Bf66946 binds to the surface of Gram-positive bacteria *Staphylococcus aureus* and *Streptococcus pneumonia* via a couple of acidic residues at the concave. In addition, the closest homolog of Bf66946 is highly expressed in the potential immune organ gill of *Branchiostoma belcheri*. Altogether, our findings provide the first structural evidence for the emergence of VLR-like molecules in the basal chordates.

Exploration of the origin and evolution of vertebrate adaptive immune system (AIS) is helpful to better understand the molecular mechanism of auto-immunity diseases. The B-cell and T-cell antigen receptors (BCRs and TCRs) based immunity has long been regarded as a unique character of AIS. It presents only among jawed vertebrates which evolved in Silurian about 430 million years ago when the first jawed fish emerged^1^. Recent discovery of novel antigen receptors termed variable lymphocyte receptors (VLRs) in jawless fishes including lamprey and hagfish reveals that AIS has evolved in a deeper time about 500 million years ago in Late Cambrian when the first jawless fish appeared^2^. The VLRs in jawless fishes are composed of tandem arrays of leucine-rich repeats (LRRs) that might be diversified by a gene conversion mechanism involving lineage-specific cytosine deaminases^3^,^4^. Three VLR genes have been identified in lampreys and hagfish (VLRA, VLRB and VLRC)^2^,^3^,^5^,^6^,^7^. VLRA is displayed on the surface of T-like lymphocytes that develop in a thymus-like tissue at the tip of gill filaments, whereas VLRB is expressed and secreted as a multivalent protein in B-like lymphocytes that develop in hematopoietic tissues^8^,^9^. In contrast, VLRC is expressed in a distinct lymphocyte lineage that may be equivalent to mammalian γδ T cells^10^,^11^. The discovery of VLR-based AIS has provided new insight into the emergence of AIS, but it is still uncertain whether AIS exists in invertebrates.

Genome sequencing of the amphioxus *Branchiostoma floridae*, a living representative of basal chordates in the evolutionary proximity to the ancestor of vertebrates, provides crucial insights into the basal chordate condition^12^. Moreover, the amphioxus genome exhibits considerable synteny with vertebrate genomes^13^,^14^, making amphioxus a valuable model for understanding the development and evolution of vertebrate immunity. In the past decades, a series of findings indicated that amphioxus also possesses some homologs of the basic components of TCR/BCR-based AIS that have been previously identified only in jawed vertebrates. These components include the lymphocyte-like cells^15^, a proto major histocompatibility complex region^16^ and homolog of recombination-activating gene 1^17^, as well as two immunoglobulin superfamily members (V region-containing chitin-binding proteins, V and C domain-bearing protein)^18^,^19^,^20^. Nevertheless, it remains unknown if any components of AIS specifically encoded by jawless vertebrates, such as VLRs, exist in amphioxus.

VLRs are featured with a LRR domain of multiple LRR modules and an invariant stalk region rich in threonine and proline residues^2^. Genomics studies have revealed that the amphioxus *B. floridae* genome contains a great number of LRR-containing gene models, of which 948 contain LRR only, a feature of VLRs^21^. Such a huge arsenal of LRR-only models indicated that VLR-like proteins may evolve in amphioxus. Thus, we performed domain analysis using the Interpro website (http://www.ebi.ac.uk/interpro/) and obtained nine hypothetical VLR-like candidates. To investigate whether these putative VLRs possess any characteristics of VLRs, two proteins Bf66946 and Bf289081 were applied to further structural and functional studies. Finally, only the protein Bf66946 yielded a crystal structure at 1.79 Å resolution, which indeed shares an overall structure quite similar to VLRs. Like other VLRs, Bf66946 specifically adheres to the Gram-positive bacteria *Staphylococcus aureus* and *Streptococcus pneumonia* using the concave surface as the antigen-binding region. Moreover, tissue-specific expression profiling revealed that Bb24355, the closest homolog of Bf66946 in *Branchiostoma belcheri*, is highly expressed in the potential immune organ gill. These findings indicated that VLR-like molecules and VLR-based adaptive immunity might have evolved at a very early history of the chordate evolution.

## Results and Discussion

### Bioinformatics analyses of putative VLR-like proteins from amphioxus *B. floridae*

The extracellular domain of VLRs usually comprise of several modules: an N-terminal LRR-capping module (LRRNT), the first LRR (LRR1), up to nine 24-residue variable LRRs (LRRVs), a variable end LRR (LRRVe), a truncated LRR designated the connecting peptide (CP), and a C-terminal LRR-capping module (LRRCT)^2^. Searching against the database of Interpro website (http://www.ebi.ac.uk/interpro/), we obtained 207 unique outputs containing LRRNT module (IPR000372) and 237 unique ones containing LRRCT module (IPR000483) in amphioxus *B. floridae*. Among these outputs, 101 unique proteins contain both LRRNT and LRRCT. As VLRs are LRR-containing proteins without any other domain^21^, we subsequently performed domain analysis and then obtained 40 proteins for further analyzed. As the LRRV modules conservatively possess a consensus sequence of XLXXLXXLXLXXNXLXXLPXXXFX (where X stands for any amino acid)^22^, nine hypothetical proteins were finally selected as VLR-like candidates. Finally, the coding sequences of the two shortest proteins, Bf66946 (GenBank accession No. EEN54567.1) and Bf289081 (GenBank accession No. EEN46450.1), were synthesized by Sangon Biotech (Shanghai) for further investigation.

### Overall structure of Bf66946

We applied both the recombinant Bf66946 and Bf289081 to crystallization trials; however, only the crystal structure of Bf66946 was successfully determined at a resolution of 1.79 Å in the space group P2_1_2_1_2_1_ (Table 1). Each asymmetric unit contains two molecules with a total buried interface area of 567 Å^2^, which is not sufficient to maintain a stable dimer. In fact, gel-filtration chromatography also indicated that Bf66946 exists as a monomer in solution.

**Figure 1:**
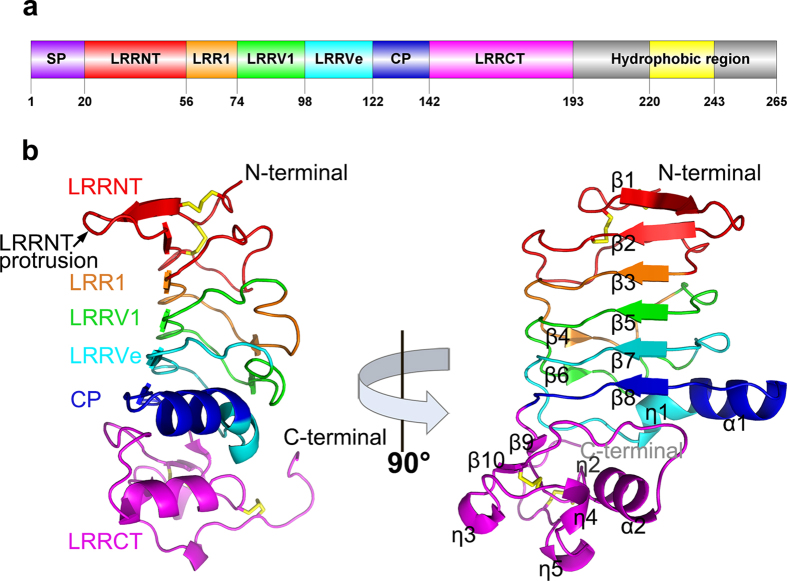
The overall structure of Bf66946. (**a**) A schematic diagram of Bf66946 drawn by Domain Graph v.1.0^45^. The regions from left to right are marked in different colors: signal peptide (SP), N-terminal LRR-capping module (LRRNT), the first LRR (LRR1), the variable LRR (LRRV), the variable end LRR (LRRVe), connecting peptide (CP), C-terminal LRR-capping module (LRRCT), and the hydrophobic region. (**b**) Ribbon diagrams of Bf66946. Disulfide bonds are shown in yellow sticks.

The overall structure of Bf66946 (residues Cys21–Asp202) adopts a crescent-shaped solenoid conformation, rich of conserved leucine and isoleucine residues involved in formation of the hydrophobic core, which is typical for the LRR family^23^,^24^. The structure contains a 36-residue LRRNT, an 18-residue LRR1, two 24-residue LRRVs (LRRV1 and LRRVe), a 20-residue CP and a 51-residue LRRCT (Fig. 1a). The LRRNT and LRRCT cap the ends of the solenoid to protect the hydrophobic core. The inner concave surface of Bf66946 is formed by a β-sheet made up of six β-strands (β1, β2, β3, β5, β7 and β8), with β1 anti-parallel to the remaining β-strands that are arranged in a parallel fashion (Fig. 1b). The outer convex surface is composed of more diverse secondary structure elements, including several loops of different length, one η-helix, two α-helices, and three 3_10_-helices (Fig. 1b). Notably, the 20-residue CP in Bf66946 folds into an α-helix that shares no identity with the 13-residue CP of previously identified VLRs.

### Bf66946 resembles VLRC rather than VLRA or VLRB

To explore the molecular function of Bf66946, we compared this structure against structures deposited in the Protein Data Bank using the Dali server (http://www.ebi.ac.uk/dali/fssp/), and obtained three closest homologs (PDB code 3TWI, 3G3A, 2R9U), all of which are VLRs from the lamprey *Petromyzon marinus*. Structural comparisons showed that the Cα atoms from LRRNT to LRRCT of Bf66946 could be well superimposed onto these three VLRs (Supplementary Fig. 1a). The hydrophobic core of Bf66946 is flanked by the cysteine-rich N- and C-terminal modules called LRRNTs and LRRCTs, respectively (Fig. 1), resembling the previously identified VLRs^2^,^5^,^6^,^25^. Moreover, the N- and C-terminal caps of Bf66946 also have four strictly conserved disulfide bonds presumably important for the structural integrity (Supplementary Fig. 1b): two within LRRNT (Cys21-Cys27, Cys25-Cys39) and the other two within LRRCT (Cys146-Cys171, Cys148-Cys192). These disulfide pairs are structurally equivalent to those previously reported VLRs^26^. In sum, the similar overall structure and key features strongly suggested that Bf66946 should represent the first characterized VLR-like protein in amphioxus.

**Figure 2:**
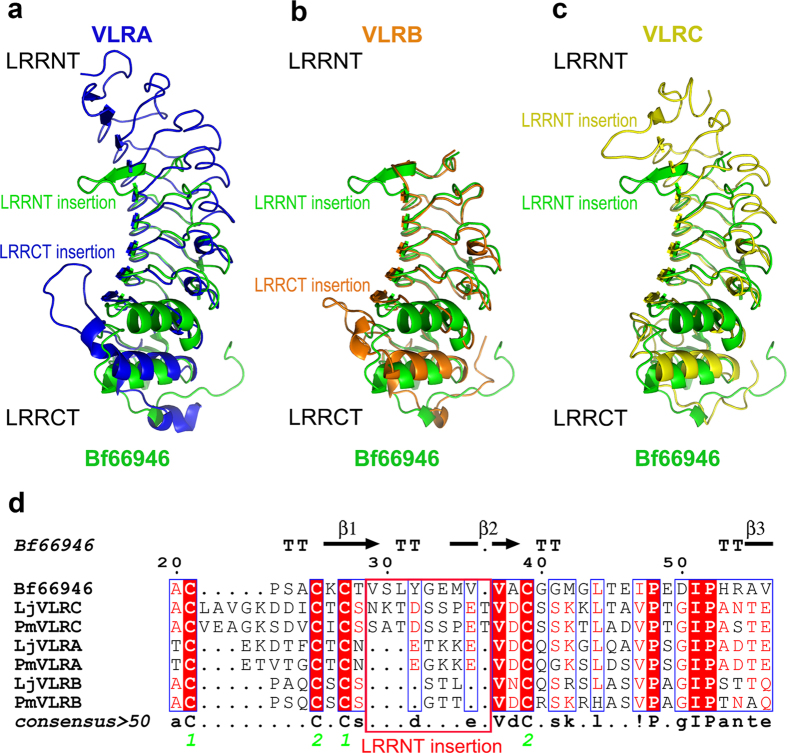
Comparisons between Bf66946 and representative VLRs. (**a**–**c**) Structural comparison of Bf66946 against representative VLRs. The crystal structures of lamprey VLRA (in blue, PDB ID: 3M18), VLRB (in orange, PDB ID: 3TWI), and VLRC (in yellow, PDB ID: 3WO9) were retrieved from Protein Data Bank (PDB). (**d**) Structure-based sequence alignment of LRRNT modules among Bf66946 and representative VLRs. All sequences were downloaded from the GenBank database (http://www.ncbi.nlm.nih.gov/genbank). PmVLRA (ACT31456.1), PmVLRB (ABY47911.1), PmVLRC (AGJ51114.1) from sea lamprey (Pm, *Petromyzon marinus*) and LjVLRA (BAJ14924.1), LjVLRB (BAJ14925.1), LjVLRC (BAJ14926.1) from Arctic lampery (Lj, *Lampetra japonica*) are shown in the alignment. White characters on a red background show strictly conserved residues. Residues that are well conserved are drawn in red and the remaining residues are black. The LRRNT protrusion is boxed with red lines. The strictly conserved bonded cysteine pairs are labeled with green numbers 1 and 2.

In consequence, the structure of Bf66946 was superimposed onto three types of VLR, termed VLRA (PDB ID: 3M19, RMSD = 1.87 Å for 138 Cα atoms)^27^, VLRB (PDB ID: 3TWI, RMSD = 1.81 Å for 156 Cα atoms)^28^ and VLRC (PDB ID: 3WO9, RMSD = 1.88 Å for 139 Cα atoms)^29^, respectively. The LRRCT region in lamprey VLRA and VLRB molecules contains a stretch of amino acids that displays marked variations in length, amino acid composition and secondary structure (Fig. 2a,b). This stretch of residues, known as a hyper-variable insertion, can form a loop structure or protrusion important for antigen recognition in both lamprey VLRA and VLRB molecules^27^,^28^,^30^,^31^. However, a corresponding insertion stretch of Bf66946 exists at LRRNT, but not LRRCT. This insertion of residues 29–36 covers a part of two strands β1–2 and the connecting loop (Fig. 2d). In fact, the N-terminal cap of lamprey VLRC also has a long loop protruding towards the concave surface (Fig. 2c); and moreover, the stretch of residues constituting LRRNT protrusion is highly conserved in length and amino acid composition in all known lamprey VLRC sequences^29^,^32^. Structure-based sequence alignment showed the LRRNT modules of VLRA and VLRB are too short to form protrusions (Fig. 2d). Previous studies also revealed all VLRA molecules and most VLRB molecules lack the potential to form a corresponding protrusion at LRRNT region^29^,^32^. Altogether, lamprey VLRA and VLRB possess an insertion in LRRCT, whereas Bf66946 and lamprey VLRC possess a protruding loop in LRRNT, suggesting that Bf66946 is more likely a putative VLRC rather than either VLRA or VLRB.

Notably, among the nine VLR-like candidates that we initially selected, three proteins (Bf66946, Bf289081 and Bf212402) may contain an LRRNT insertion, whereas none of them has an LRRCT insertion (Supplementary Fig. 2). However, the numbers of LRR repeats in these candidates vary a lot, from 2 repeats in Bf66946 to 9 repeats in Bf69561 and Bf104848.

### Bf66946 binds to Gram-positive bacteria via a negatively-charged patch at the concave surface of solenoid structure

**Figure 3:**
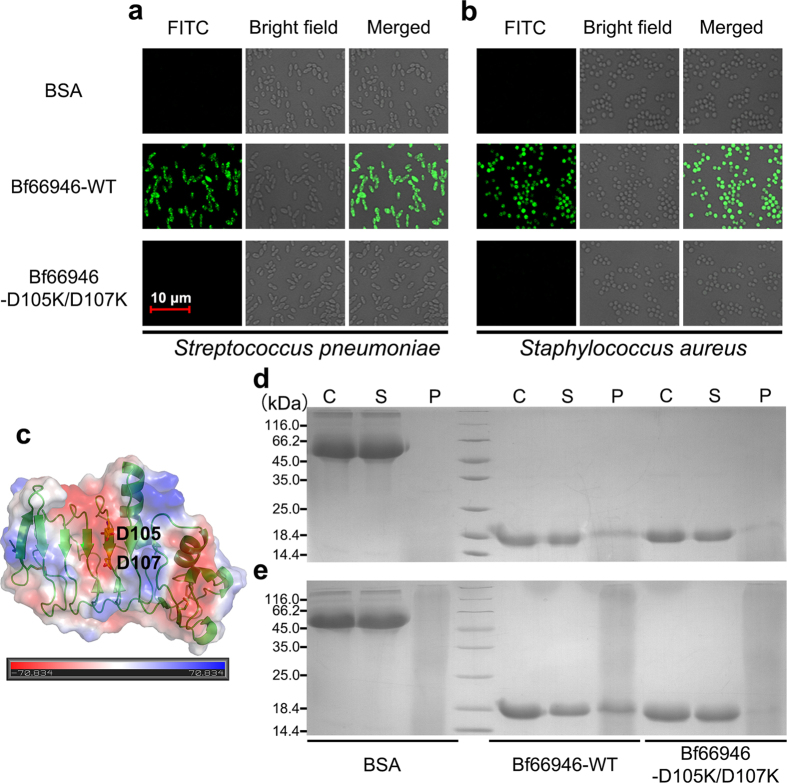
Binding activity assays. The bacterial binding assays of Bf66946 and its double mutant Bf66946-D105K/D107K towards (**a**) *S. pneumonia*, (**b**) *S. aureus*. (**c**) The electrostatic potential of Bf66946. Electrostatic potential analysis showed a negatively-charged patch in the concave of the LRR solenoid structure. The two acidic residues Asp105 and Asp107 are labled and shown in sticks. Fractions from the peptidoglycan binding assays with (**d**) *S. pneumonia* or (**e**) *S. aureus* peptidoglycan using wild-type Bf66946, mutant Bf66946-D105K/D107K and BSA were analysed in SDS-PAGE. In each gel: **C**, respective ‘input proteins′ (BSA, wild-type Bf66946 and mutant Bf66946-D105K/D107K); **S**, supernatant from binding mixture (unbound proteins); **P**, sample of the pellet from the binding mixture (bound proteins).

Jawless vertebrate AIS employs VLRs as antigen receptors to recognize invaded pathogens, such as viruses and bacteria. As Bf66946 is similar to VLRs, it should recognize some ligands displayed on the surface of pathogens. Therefore, we detected the bacterial binding activity of Bf66946 towards some bacteria, using bovine serum albumin (BSA) as the negative control. The results showed that Bf66946 specifically adheres to Gram-positive bacteria *S. aureus* and *S. pneumoniae*, but not Gram-negative bacteria *Escherichia coli* and *Salmonella enterica* (Fig. 3a,b and Supplementary Fig. 3a,b).

To identify the precise ligand-binding site of Bf66946, we compared the binding capacity of Bf66946 with the mutants. The concave surface of the LRR solenoid structure has been proved to be the antigen-binding surface of VLRs^22^,^27^,^28^,^29^,^30^,^31^, indicating that the concave surface of Bf66946 is most likely the major ligand binding region. Indeed, the electrostatic potentials analysis displays a negatively-charged patch within the concave surface of the LRR solenoid structure (Fig. 3c), implying it might recognize a positively-charged ligand. A couple of mutants related to the negatively-charged region within the concave surface were designed, such as Bf66946-Y32A/E34A (located at the LRRNT insertion), Bf66946-D105K/D107K (located at the β-strand of LRRVe). These mutants were cloned, overexpressed and purified by the same way as the wild-type Bf66946, and circular dichroism spectra confirmed these mutatants are well folded as the wild-type protein (Supplementary Fig. 4). Subsequent bacterial binding assays revealed that Bf66946-D105K/D107K completely lost the binding capacity towards *S. aureus* and *S. pneumonia* (Fig. 3a,b), whereas the double mutation Y32A/E34A did not alter the binding capacity. Thus, we identified that the residues Asp105 and Asp107 at the concave are key to recognition of Gram-positive bacteria (Fig. 3c). These results suggested that Bf66946 is indeed VLR-like protein using the concave surface of the LRR solenoid structure to recognize antigens.

As we know, the Gram-positive bacteria differ from the Gram-negative bacteria with the peptidoglycan exposed. In fact, after treatment with 50 mM EDTA for 10 min, the Gram-negative bacteria *E. coli* and *S. enterica* also possess the binding affinity towards Bf66946 (Supplementary Fig. 3a-d). Moreover, *in vitro* binding assays also indicated Bf66946 specifically binds to the peptidoglycan purified from both Gram-positive and negative bacteria; however, the Gram-negative bacterial peptidoglycan has a much lower binding affinity (Fig. 3d,e and Supplementary Fig. 3e,f).

### The closest homolog of Bf66946 is specifically expressed in the potential immune organ gill of *B. belcheri*

**Figure 4:**
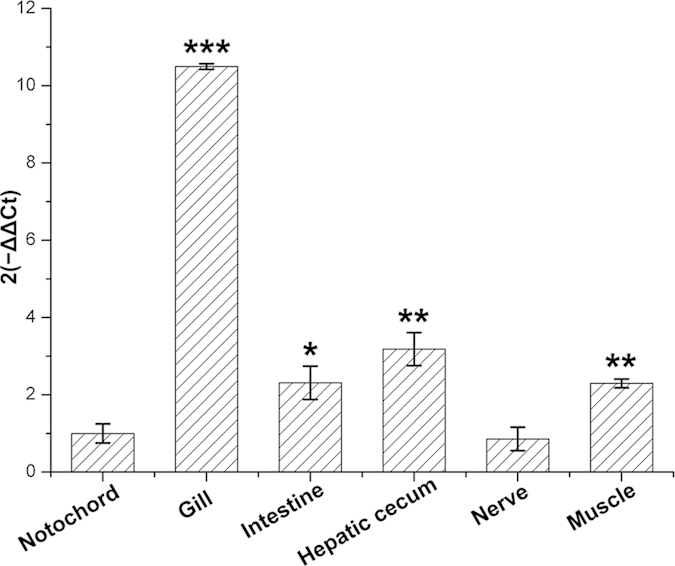
Relative expression levels of Bb24355 gene in various tissues of *B. belcheri* by qRT-PCR. 2^−ΔΔCt^ values (normalized to endogenous control 18S) represented the expression of Bb24355 gene in various tissues (notochord, gill, intestine, hepatic cecum, nerve and muscle) of *B. belcheri*. Bars represent the average results of triplicate reactions for each tissue. Error bars represent standard error of the mean (SEM) among three biological replicates. *, ** and *** indicate significant expression level difference versus notochord as control at *p* < 0.05, *p* < 0.01 and *p* < 0.001, respectively.

Due to the difficulty of raising the amphioxus *B. floridae* in the laboratory, we alternatively detected the expression profile of the homolog of Bf66946 in *B. belcheri*. Searching against the database of Transcriptome from v4.0 Online (data unpublished), Bbelcheri_v4_101980.1|cds_24355 (Bb24355) was identified as the closest sequence relative of Bf66946. Sequence comparisons showed that Bf66946 is of 86% sequence identity to that of Bb24355 (Supplementary Fig. 5). The method of 2^−ΔΔCt^ for histogram chart was used to analyse the gene expression levels of Bb24355 in various tissues (notochord, gill, intestine, hepatic cecum, nerve and muscle), respectively. Each tissue has a 2^−ΔΔCt^ value to represent the gene expression level of Bb24355. The expression level of Bb24355 in gill was the highest and significantly higher than that in other tissues (Fig. 4). Moreover, a cluster of cells with morphological similarity to vertebrate lymphocytes has been identified in amphioxus gill^15^, suggesting the gill is a potential immune organ.

In summary, we identified Bf66946 from *B. floridae* as an analog of variable lymphocyte receptor VLRC using bioinformatics analysis and structural comparison combined with *in vitro* bacterial binding assays. This is the first characterized VLR-like protein in amphioxus, suggesting the emergence of VLR-based AIS in basal chordates. However, further investigations are needed to define the bona fide substrate of Bf66946, and other basic components of VLR-based AIS.

## Methods

### Protein expression, purification and crystallization

The coding sequence of Bf66946 was synthetized by Sangon Biotech (Shanghai). Recombinant Bf66946 containing residues 21 to 202 was cloned into the NdeI/XhoI site of pET29b-derived expression vector and expressed as inclusion bodies in *E. coli* strain BL21 (DE3) (Novagen, Madison) using 2 × YT (yeast extract and tryptone) culture medium with 30 μg/ml kanamycin. Bacteria were grown at 37 °C to an absorbance of 0.8 at 600 nm and then induced with 0.2 mM isopropyl-β-D-1-thiogalactopyranoside for an additional 4 hr. The bacteria were harvested by centrifugation and resuspended in buffer A (50 mM Tris-HCl pH 8.0, 0.1 M NaCl and 2 mM EDTA). After sonication for 20 min, inclusion bodies were collected and washed with buffer B (50 mM Tris-HCl pH 8.0, 0.1 M NaCl, 2 mM EDTA and 0.5% (v/v) Triton X-100), then solubilized in denaturing buffer (50 mM Tris-HCl pH 8.0, 10 mM DTT, 5 mM EDTA, 8 M urea). For *in vitro* refolding, denaturing buffer containing proteins were diluted into refolding buffer (0.8 M arginine, 50 mM Tris-HCl pH 8.0, 2 mM EDTA, 3.7 mM cystamine, 6.6 mM cysteamine) to a final concentration of 10 mg/l and stirred for 72 hr at 4 °C. Afterwards, the refolding mixture was concentrated, dialyzed against 50 mM MES pH 6.0 at 4 °C overnight to remove arginine. After centrifugation at 12,000 × g for 30 min, the supernatant containing the soluble target protein was dialyzed against 20 mM Tris-HCl pH 8.0, and applied to a QHP column (GE Healthcare, UK) followed by gel filtration with a HiLoad 26/60 Superdex 75 prep-grade column (GE Healthcare, UK) for further purification. Fractions containing the target protein were pooled and concentrated to 10 mg/ml for crystallization. The concentration of protein was measured using absorbance spectroscopy at 280 nm (OD-1000 Spectrophotometer, One Drop). The purity of protein was assessed by electrophoresis and the protein samples were stored at −80 °C.

Site-directed mutagenesis was performed using the PCR-based site-directed mutagenesis with the plasmid encoding the wild-type Bf66946 as the template. The mutants were expressed, purified and stored in the same manners as the wild-type Bf66946.

Selenomethionine (SeMet)-labeled Bf66946 (Se-Bf66946) was expressed in *E. coli* strain B834 (DE3) (Novagen). Transformed cells were inoculated into LB medium and incubated at 37 °C. The cells were harvested when the A_600 nm_ reached 0.2 and were then washed twice with M9 medium. The cells were then cultured in SeMet medium (M9 medium with 25 μg/ml L-SeMet and 50 μg/ml other essential amino acids) containing 30 μg/ml kanamycin to the absorbance of 0.8 at 600 nm. The remaining steps of protein expression, purification and storage of Se-Bf66946 were the same as those for native Bf66946.

### Crystallization, Data Collection and Processing

Se-Bf66946 was concentrated to 10 mg/ml by ultrafiltration (Millipore Amicon) for crystallization. Screening for the crystallization conditions of Se-Bf66946 was performed using screening kits of Crystal Screen I and II, Index, Grid screens and SaltRx (Hampton Research) with the hanging drop vapor-diffusion method in 96-well plates at 16 °C. Crystals of Se-Bf66946 were grown with the initial condition of mixing 1 μl protein sample with an equal volume of reservoir solution (15% (w/v) polyethylene glycol 8000, 0.1 M Bis-Tris pH 6.2) against 0.5 ml reservoir solution. Typically, crystals appeared in 1–2 days and reached the maximum size in one week. The crystals were transferred to cryoprotectant (reservoir solution supplemented with 25% glycerol) and flash-cooled with liquid nitrogen. SeMet-derivative data sets were collected from single crystal at 100 K in a liquid-nitrogen-gas stream using an ADSC Q315r CCD detector (Area Detector Systems Corporation) on beamline BL17U at Shanghai Synchrotron Radiation Facility (SSRF). The data were collected at a radiation wavelength of 0.97915 Å. All diffraction data were indexed, integrated, and scaled with the program HKL2000^33^.

### Structure determination and refinement

The crystal structure of Bf66946 was determined by the single-wavelength anomalous dispersion (SAD) phasing method using data from a single SeMet-substituted protein crystal to a maximum resolution of 1.79 Å. The AutoSol program from PHENIX^34^ was used to locate the selenium atoms and to calculate the initial phases. Automatic model building was carried out using AutoBuild in PHENIX^34^. Refinement was carried out using the maximum likelihood method implemented in REFMAC5^35^ as part of CCP4i^36^ program suite and the model was rebuilt interactively using the program COOT^37^ until the free R-factor converged. The final model was evaluated with MOLPROBITY^38^ and PROCHECK^39^. Crystallographic parameters were listed in Table 1. Structural superpositions were performed by the program Superpose Molecules as implemented in CCP4i program suite.

### *In vitro* bacterial binding assays

Labeling of protein was performed with fluorescein isothiocyanate (FITC, Sigma-Aldrich) according to a conventional protocol. Briefly, the protein sample at a concentration of 5 mg/ml was prepared in 0.1 M sodium carbonate buffer, pH 9.0. Then 50 μl of FITC solution (1 mg/ml FITC in anhydrous dimethyl sulfoxide solution) was added into each 1 ml of protein solution, very slowly in 5 μl aliquots while gently and continuously stirring the protein solution. The reaction was incubated in the dark for 8 hr at 4 °C. After that, a final concentration of 50 mM NH_4_Cl was added and incubated for 2 hr at 4 °C to terminate the reaction. The desalting column (GE Healthcare) was used to separate the unbound FITC from the conjugate with phosphate buffered saline (PBS) solution (10 mM Na_2_HPO_4_, 1.8 mM KH_2_PO_4_, 137 mM NaCl, 2.7 mM KCl. pH 7.4), and fractions containing the conjugate were pooled and stored in PBS in a lightproof container at 4 °C. The ratio of fluorescein to protein of the product was estimated by measuring the absorbance at 495 nm and 280 nm.

*E. coli* BL21 (DE3) strain (Novagen), *S. enterica*, *S. aureus* (capsular type S strain Reynolds) and *S. pneumonia* Strains (rough, type 6; LytA knockout) were used in this assays. *E. coli*, *S. enterica* and *S. aureus* were grown at 37 °C using 2 × YT culture medium, whereas *S. pneumonia* was cultured in 2 × THY (3% Todd-Hewitt Broth, 0.2% Yeast Extract) under aerobic conditions (37 °C, 5% CO_2_). When the absorbance at 600 nm of bacteria reached about 0.6, bacterial strains were harvested by centrifugation at 1,500 × g for 10 min. After washed three times, the bacterial pellets were resuspended in PBS and adjusted to an optical density (OD) of 0.6 (1 cm light path) at 600 nm.

In each reaction, the pellets of 400 μl bacteria were resuspended with the FITC-labeled protein and incubated in the dark for 1 hr at 25 °C with shaking gently. Afterwards, the bacterial pellets were washed in PBS for five times to remove the unbound protein. The bacterial samples were observed and photographed with con-focal laser scanning microscope (Zeiss LSM 710).

### Peptidoglycan purification

Peptidoglycan from Gram-positive/negative bacteria was purified as described previously^40^ with modifications. Briefly, Cells of a 2-L culture were harvested at an OD_620_ of 0.5 by centrifugation for 30 min at 4 °C and 7,500 × g. The cell pellet was resuspended in 40 ml of ice-cold 50 mM Tris–HCl (pH 7.0). The cell suspension was introduced dropwise into a flask with 150 ml of boiling 5% sodium dodecyl sulfate (SDS) solution. The solution was boiled for another 30 min. The cell suspension was pelleted by ultracentrifugation at 25 °C for 30 min at 130,000 × g. The pellet was washed twice with 30 ml of 1 M NaCl and repeatedly with deionized water (dH_2_O) until it was free of SDS, confirmed by a previously published assay. The pellet was resuspended in 20 ml of 100 mM Tris–HCl (pH 7.5) containing 20 mM MgSO_4_. DNase A and RNase I were added to final concentrations of 10 and 50 μg/ml, respectively, and the sample was stirred for 2 hr at 37 °C. CaCl_2_ (10 mM) and trypsin (100 μg/ml) were added, and the sample was stirred for 18 hr at 37 °C. Then, the sample was incubated for 15 min at 80 °C to inactivate the enzymes. The cell wall was recovered by centrifugation for 45 min at 130,000 × g at 25 °C, resuspended with 20 ml of 8 M LiCl, and incubated for 15 min at 37 °C. After another centrifugation (see above), the pellet was resuspended in 20 mM EDTA (pH 7.0) and incubated at 37 °C for 1 hr. The cell wall was washed with dH_2_O before finally being resuspended in 2 to 4 ml of dH_2_O prior to lyophilization. Cell wall samples were stored at −20 °C.

Next, for Gram-positive bacteria only, 5 mg of cell wall from was stirred with 48% hydrofluoric acid (HF) for 48 hr at 16 °C in an ultracentrifuge tube made of polyallomer tightly closed by Parafilm. The volatile, aggressive, and toxic HF was handled with great caution to avoid any contact with the skin and spillage. The peptidoglycan was recovered by centrifugation (45 min, 130,000 × g, 4 °C) and washed with ice-cold dH_2_O, 100 mM Tris–HCl (pH 7.0), and then twice with dH_2_O. The pellet of peptidoglycan was resuspended in 1 ml dH_2_O and stored at 4 °C.

### Peptidoglycan binding assays

Peptidoglycan binding of the recombinant proteins was assessed using an assay described previously^41^ with modifications. In short, the peptidoglycan was mixed at 10 mg/ml concentration with the tested proteins (Bf66946-WT and Bf66946-D105K/D107K) at 1 mg/ml concentration in PBS and incubated for 30 min at 25 °C to allow binding. BSA were used as negative controls. After incubation, the mixtures were centrifuged at 12,000 × g for 10 min to sediment the peptidoglycan, and the supernatants were collected. The peptidoglycan pellet fraction was washed with PBS for three times, and dissolved in SDS loading buffer. Samples from the supernatant (unbound protein) and the resuspended pellet (bound protein) both were analyzed in SDS-PAGE and stained with Coomassie Brilliant Blue.

### Circular Dichroism (CD) spectroscopy

CD spectra (250 to 190 nm) of protein (4 μM) in 0.1 M sodium phosphate buffer (pH 8.0) were recorded at 25 °C on a Jasco J810 Spectrophotometer using a quartz cuvette with a 0.1 cm path length and instrument setting of 1 nm band-width, 0.5 nm data-interval and 30 second averaging time. Spectra were recorded in triplicate, averaged, and background subtracted^42^.

### Animal cultivation

The adults of amphioxus *B. belcheri* were obtained from Zhanjiang, Guangdong Province, China, cultured at 24–25 °C with air-pumped circulating seawater, and fed with green alga *Chlorella*.

### Quantitative real-time PCR

Total RNA samples were extracted from distinct tissues (every kind of tissues was collected from five amphioxus) of *B. belcheri* using Trizol Reagent (Invitrogen, Carlsbad, CA, USA) according to the manufacturer’s protocols. The total RNA samples were then treated with RNase-free DNase (TaKaRa, Dalian, China) using a standardized protocol. Then 1 μg of total RNA were reverse transcribed to cDNA with oligodT (TaKaRa, Dalian, China). *B. belcheri* VLR-like homolog (Bb24355) was identified by using *B. floridae* Bf66946 as the BLAST query against the database of Transcriptome from v4.0 Online (data unpublished). For the analysis of Bb24355, quantitative real-time PCR was performed on an Applied Biosystems 7300 Sequence Detection System (Applied Biosystems, Foster City, CA, USA) using a SYBR Green RT-PCR kit (DRR420A, TaKaRa, Dalian, China). Amplification reactions were incubated in a 96-well plate with a thermal cycling profile of beginning at 95 °C for 30 sec, followed by 40 cycles of 95 °C for 5 sec and 60 °C for 31 sec^43^. All of the reactions were performed in triplicate. The sequences of the sense and antisense primers used for the amplification of Bb24355 gene were as follows: 5′- TGTCGCATGGCCTGGTTGG-3′ (sense), 5′- CGGAGCACACGAAGGAAGCC-3′ (antisense). The 18S RNA levels were used as internal control. Data were quantified using the 2^−ΔΔCt^ method based on Ct Values^44^.

## Additional Information

**How to cite this article**: Cao, D.-D. *et al.* Structure of a variable lymphocyte receptor-like protein from the amphioxus *Branchiostoma floridae*. *Sci. Rep.* **6**, 19951; doi: 10.1038/srep19951 (2016).

## Supplementary Material

Supplementary Information

## Figures and Tables

**Table 1:** Data collection and refinement statistics of SeMet-substituted Bf66946.

**Data collection**	
Space group	P 2_1_ 2_1_ 2_1_
Cell dimensions	
*a*, *b*, *c* (Å)	50.25, 67.87, 118.04
*α*, *β*, *γ* (°)	90.00
Resolution (Å)	50.0–1.79 (1.85–1.79)
Completeness (%)	95.4 (97.3)
*R*_merge_	0.114 (0.495)
*R*_*p.i.m*_	0.053 (0.236)
*R*_*means*_*/R*_*r.i.m*_	0.127 (0.553)
I/σ(I)	10.0 (3.6)
CC_1/2_	0.993 (0.822)
Wilson B-factor (Å^2^)	14.78
Redundancy	4.8 (4.8)
**Refinement**	
Resolution (Å)	50.0–1.79 (1.85–1.79)
No. reflections	37,098 (3,703)
*R*_work_/*R*_free_	0.178/0.202
No. atoms	
Protein	2698
Ligand	18
Water	366
*B*-factors	
Protein	16.9
Ligand	29.7
Water	27.2
R.m.s. deviations	
Bond lengths (Å)	0.007
Bond angles (°)	1.252
Ramachandran plot (residues, %)	
Most favored (%)	97.7
Additional allowed (%)	2.3
Outliers (%)	0
PDB entry	4XSQ

Values in parentheses are for highest-resolution shell.
