# Supplementary material for: Structure of a variable lymphocyte receptor-like protein from the amphioxus Branchiostoma floridae
Source: Sci Rep. 2016 Jan 29;6:19951. doi: 10.1038/srep19951 (PMC4731796; doi:10.1038/srep19951)
Supplement: Supplementary Information [file srep19951-s1.pdf]

Supplementary Information

Structure of a variable lymphocyte receptor-like protein from *Branchiostoma floridae*

Dong-Dong Cao[1], Xin Liao[2], Wang Cheng[1], Yong-Liang Jiang[1], Wen-Jie Wang[1], Qiong Li[1], Jun-Yuan Chen[2*], Yuxing Chen[1*] and Cong-Zhao Zhou[1*]

[1]Hefei National Laboratory for Physical Sciences at the Microscale and School of Life Sciences, University of Science and Technology of China, Hefei Anhui 230027, China.

[2]Beihai Marine Station, Evo-devo Institute, School of Life Sciences, Nanjing University, Hankou Road 22#, Nanjing, Jiangsu, 210093, People's Republic of China

[*]Correspondence should be addressed to C.Z.Z. (email: zcz@ustc.edu.cn), Y.C. (email: cyxing@ustc.edu.cn) or J.Y.C. (email: chenjunyuan@163.net).

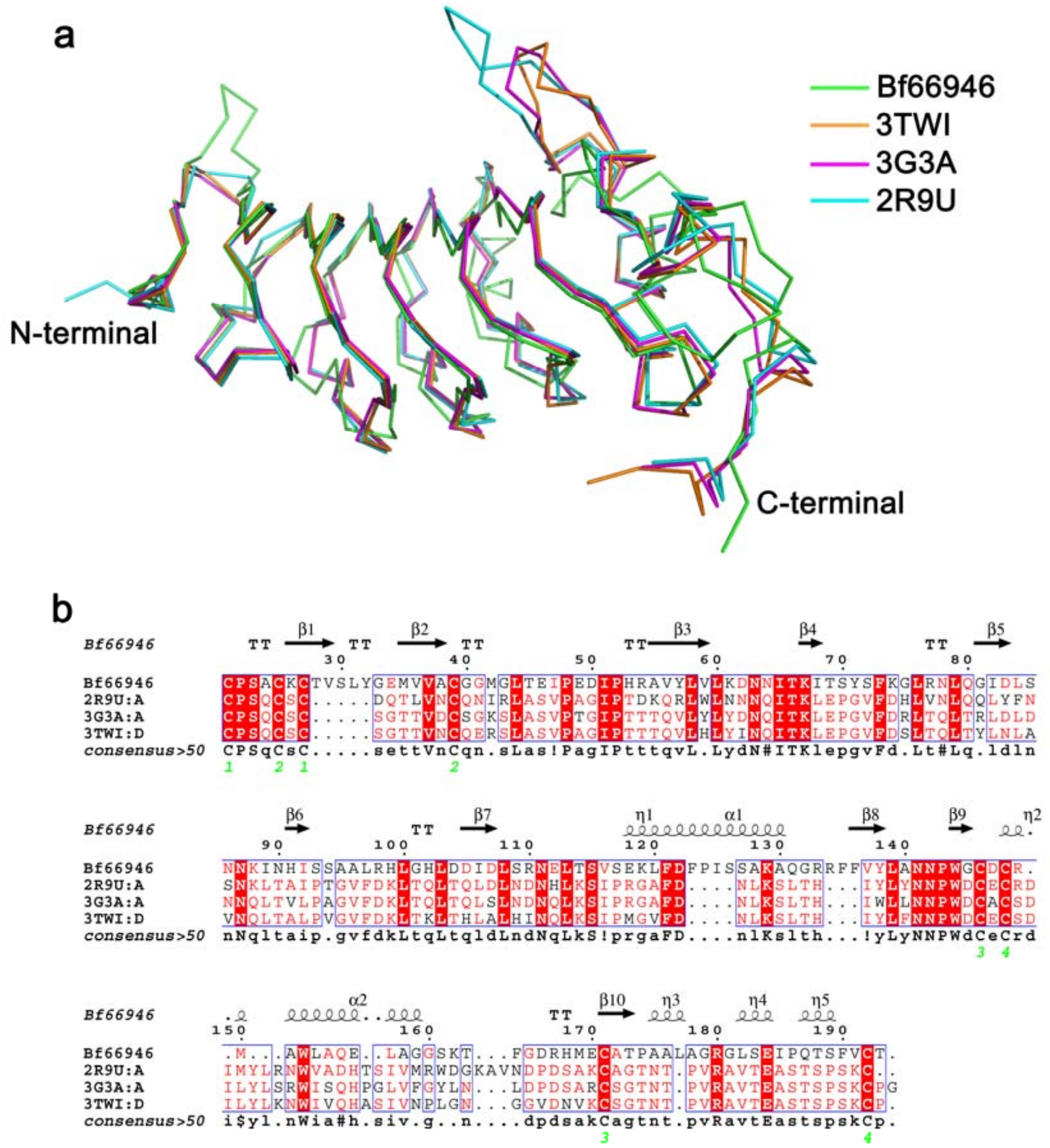

Supplementary Figure 1

Comparisons of Bf66946 with the three most resembled VLRs from Dali search. (**a**) Superposition of Bf66946 against the three VLRs with the PDB code 3TWI, 3G3A and 2R9U. (**b**) The structure-based sequence alignment of Bf66946 and the three VLRs. The strictly conserved bonded cysteine residues in the VLR structures are labeled with green numbers 1–4.

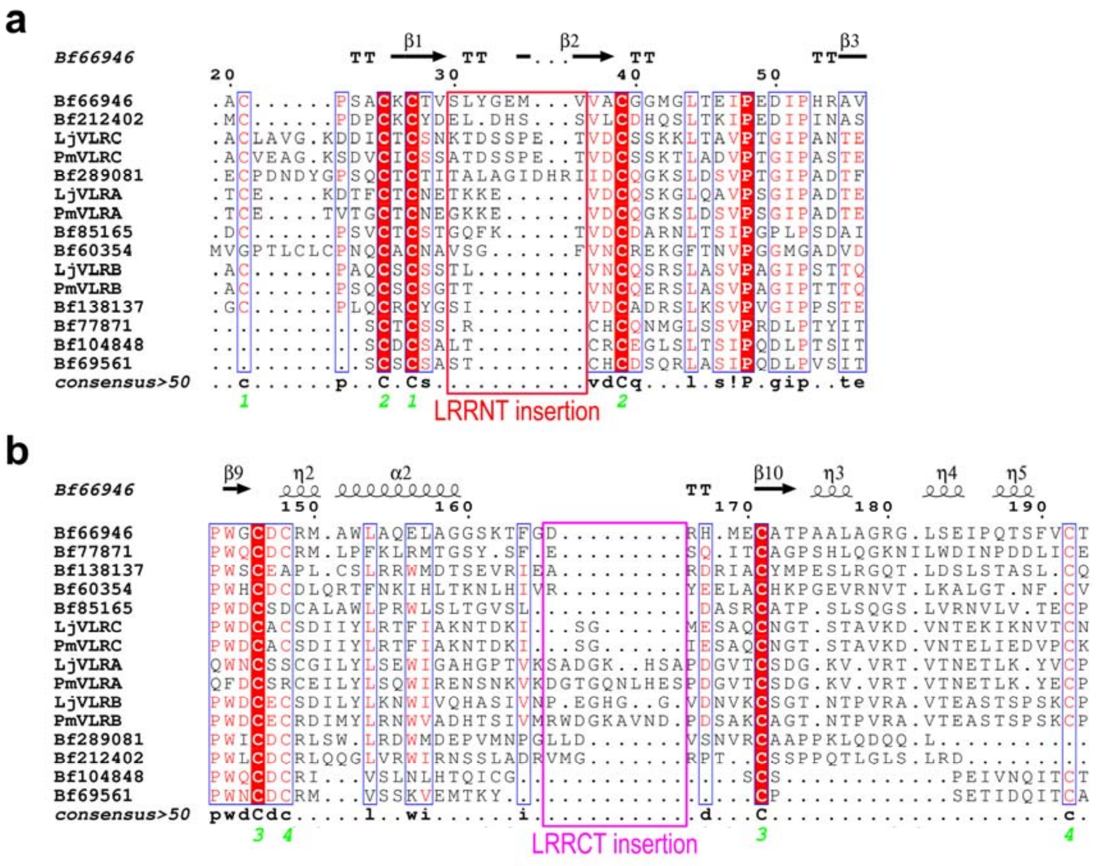

Supplementary Figure 2

Structure-based sequences alignment of nine VLR-like candidates and canonical VLRs from *Petromyzon marinus* and *Lampetra japonica* at the (**a**) LRRNT and (**b**) LRRCT, respectively. The LRRNT and LRRCT insertion is highlighted with red and magenta boxes, respectively. The strictly conserved bonded cysteine pairs are labeled with green numbers 1-4.

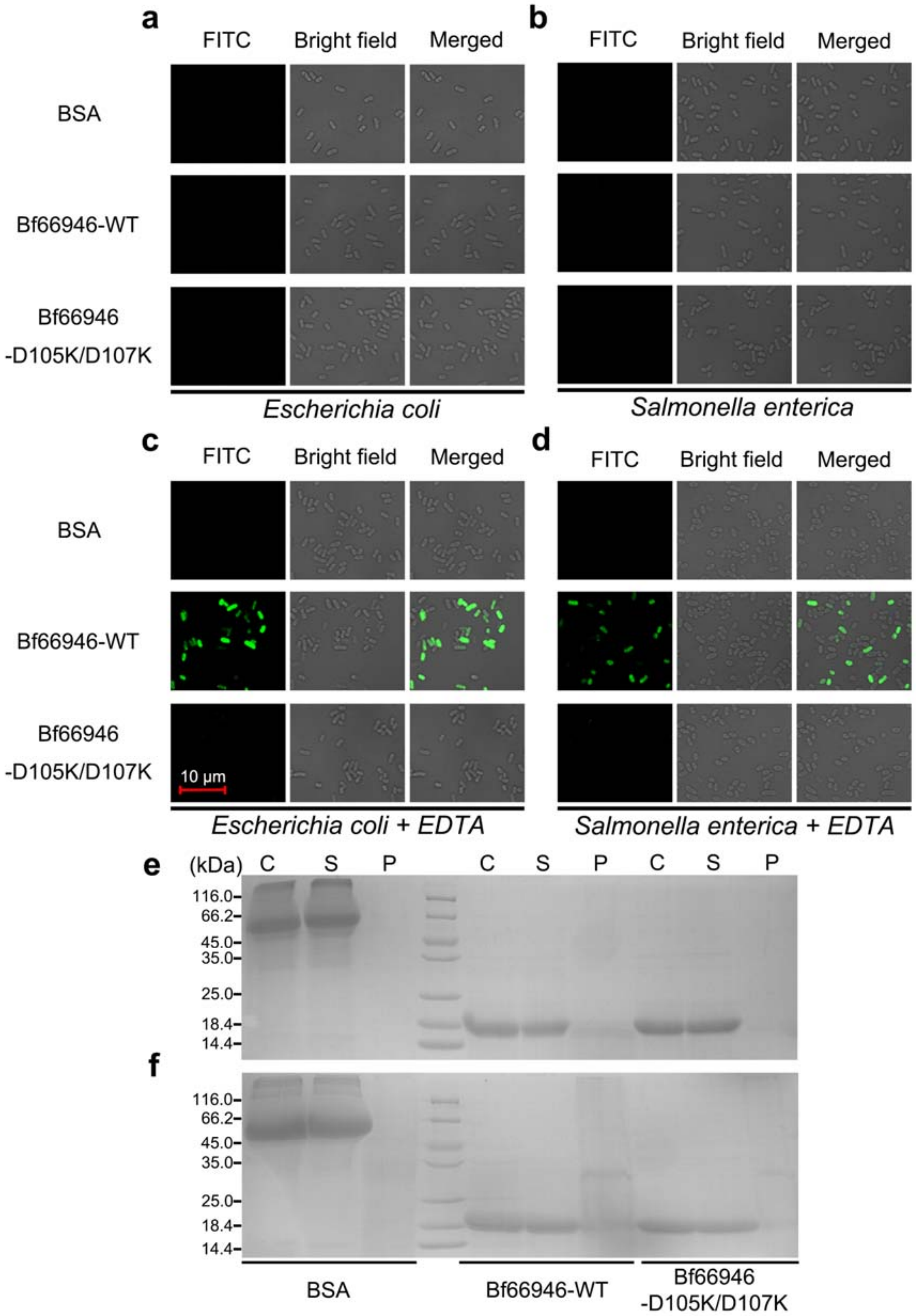

Supplementary Figure 3

Binding activity assays. The bacterial binding assays of Bf66946 and its double mutant Bf66946-D105K/D107K towards (**a**) *E. coli*, (**b**) *S. enterica*, (**c**) EDTA treated

E. coli, (**d**) EDTA treated *S. enterica*. Fractions from the peptidoglycan binding assays with (**e**) *E. coli* or (**f**) *S. enterica* peptidoglycan using wild-type Bf66946, mutant Bf66946-D105K/D107K and BSA were analysed in SDS-PAGE. In each gel: **C**, respective 'input proteins' (BSA, wild-type Bf66946 and mutant Bf66946-D105K/D107K); **S**, supernatant from binding mixture (unbound proteins); **P**, sample of the pellet from the binding mixture (bound proteins).

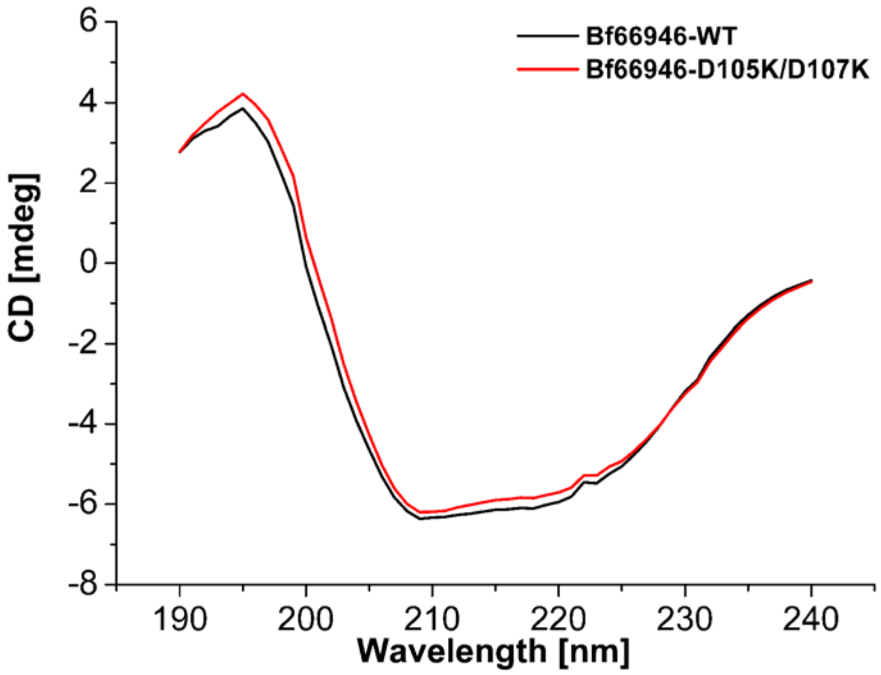

Supplementary Figure 4

Circular dichroism spectra of Bf66946-WT (black line) and Bf66946-D105K/D107K (red line) in 0.1 M sodium phosphate buffer (pH 8.0).

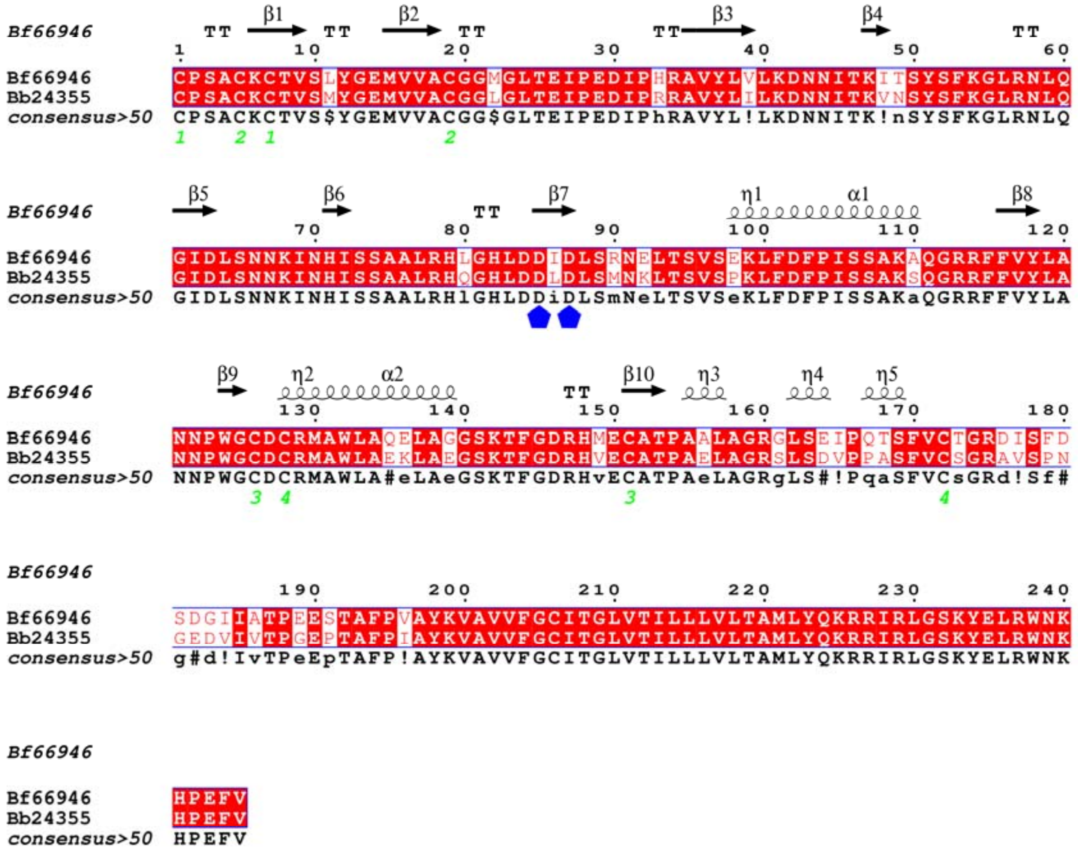

Supplementary Figure 5

Sequence alignment between Bf66946 and Bb24355. The conserved bacterial binding sites within Bf66946 and Bb24355 are shown with pentagons colored in blue. The strictly conserved bonded cysteine residues in the VLR structures are labeled with green numbers 1–4.
